# Cardioprotective Effect of Crocin Combined with Voluntary Exercise in Rat: Role of Mir-126 and Mir-210 in Heart Angiogenesis

**DOI:** 10.5935/abc.20170087

**Published:** 2017-07

**Authors:** Vajihe Ghorbanzadeh, Mustafa Mohammadi, Hassan Dariushnejad, Alireza Abhari, Leila Chodari, Gisou Mohaddes

**Affiliations:** Drug Applied Research Center - Tabriz University of Medical Sciences, Tabriz - Iran

**Keywords:** Rats, Angiogenesis Modulating Agents, Exercise, Crocus Sativus, Antioxidants, miR-126, miR-210

## Abstract

**Background:**

Crocin is reported to have a wide range of biological activities such as cardiovascular protection. Recent epidemiologic studies have shown that exercise reduces cardiovascular morbidity and mortality in the general population.

**Objective:**

The aim of this study was to evaluate the effect of crocin and voluntary exercise on miR-126 and miR-210 expression levels and angiogenesis in the heart tissue.

**Methods:**

Animals were divided into 4 groups: control, exercise, crocin, and exercise-crocin. Animals received oral administration of crocin (50 mg/kg) or performed voluntary exercise alone or together for 8 weeks. Akt, ERK1/2 protein levels, miR-126 and miR-210 expression were measured in the heart tissue. Immunohistochemical method was used to detect CD31 in the heart tissue.

**Results:**

Akt and ERK1/2 levels of the heart tissue were higher in crocin treated group and voluntary exercise trained group after 8 weeks. Combination of crocin and exercise also significantly enhanced Akt and ERK1/2 levels in the heart tissue. MiR-126, miR-210 expression and CD31 in the heart increased in both crocin and voluntary exercise groups compared with control group. In addition, combination of exercise and crocin amplified their effect on miR-126 and miR-210 expression, and angiogenesis.

**Conclusion:**

Crocin and voluntary exercise improve heart angiogenesis possibly through enhancement of miR-126 and miR-210 expression. Voluntary exercise and diet supplementation with crocin could have beneficial effects in prevention of cardiovascular disease.

## Introduction

Crocin is a bioactive constituent found in the fruits of gardenia and in the stigmas of saffron.^1^ Crocin has long been used in traditional medicine and has been reported to have various pharmacological activities, such as antioxidant, anti-cancer, anti-inflammation, anti-atherosclerotic effects, and protection against cardiovascular diseases.^2^ The cardioprotective effects of crocin has been reported in some studies that are related to modulation of endogenous antioxidant enzymatic activities and cardiac biomarkers.^3,4^ Recent evidence has indicated the protective effect of crocin on hypoxic damage of myocardial cells by elevation of vascular endothelial growth factor (VEGF), as a proangiogenic factor.^5^

Physical activity plays a critical role in metabolism, cardiovascular function, and immune function. In the last years it became evident that exercise training is a very powerful therapeutic strategy for prevention of development and progression of cardiovascular disease.^6^ Nevertheless, exhaustive exercise may be problematic, as they are stressful through production of reactive oxygen species and can cause damage to muscle tissue and other organs.^7,8^ It has been suggested that voluntary exercise may be a better model with more beneficial effects.^9^ In the animal model of voluntary exercise, the animal has free access to a running wheel and uses the wheel according to its physiological threshold for physical activity. It has been discovered that physical activity triggers extension of the capillary network or angiogenesis. This process is known to be VEGF dependent.^10^ However, the underlying mechanisms of exercise have yet to be fully elucidated.

Micro-RNAs (miRs) are small non-coding 18-25 nucleotide RNAs that play a key role in regulating gene expression by inhibiting protein translation or enhancing messenger RNA degradation.^11,12^ Their participation in cardiovascular disease has been recognized during recent years.^11-13^ MiR-126 is one of the few miRNAs that is an endothelial cell-specific miRNA and plays an essential role in neoangiogenesis. MiRNA-126 is strongly expressed in the heart endothelium and targets Sprouty-related protein-1 (Spred-1), PIK3R2, a regulatory subunit of PI3K.^14,15^ Downregulation of these targets activates survival kinases ERK and Akt and enhances the actions of vascular endothelial growth factor (VEGF).^16,17^ VEGF exerts many of its effects on angiogenesis via the Akt and ERK1/2 pathways. During developmental vasculogenesis, the Akt pathway regulates venous specification, whereas the ERK pathway regulates arterial specification.^18,19^ MiR-210 overexpression in normoxic endothelial cells stimulated the formation of capillary like structures as well as VEGF-driven cell migration.^20^ MiR-210 induction is a virtually constant feature of the hypoxic response that is important in the pathogenesis of several human diseases, such as heart disease.^21^

According to the advantage effects of crocin and voluntary exercise on diabetes that mentioned above, we hypothesized that, compared with crocin or voluntary exercise alone, 8 weeks of crocin combined with voluntary exercise in diabetic rats would produce a larger improvement in cardiovascular complications of type 2 diabetes. The present study was undertaken to clarify the effect of crocin and voluntary exercise on miR-126 and miR-210 expression in cardiac myocytes of diabetic rats.

## Methods

### Animals

Male Wistar rats (200-250g) were obtained from Tabriz medical faculty (Iran-Tabriz). Animals were housed in a room with a constant temperature of 24ºC, a relative humidity of 50%, and a 12h dark/light cycle with access to food and water ad libitum. Animals in the *exercise group* were placed in individual wheel-*cage* units while the sedentary *group*s were *housed* in normal plastic *cages*. This study was approved by the Animal Ethics Committee (document number 92197) in accordance with the instruction for the care and use of laboratory animals prepared by Tabriz University of Medical Sciences.

### Experimental design

Forty animals were randomly divided into four groups. *Ten animals were allocated* to each experimental *group a*t the beginning of the study. Group 1: Rats received NaCl 0.9 % Solution as a control group (Con). Group 2: Rats received a single dose of crocin (50 mg/kg) for eight weeks (Cro). Group 3: Rats performed voluntary exercise for eight weeks (Exe). Group 4: Animals received crocin and simultaneously performed voluntary exercise for eight weeks (Cro-Exe).

Crocin powder (Sigma, Germany) was diluted by normal saline (0.9%). Crocin was gavaged (50 mg/kg) 6 days a week for 8 weeks.^22^ In addition, NaCl 0.9 % solution was gavaged in groups 1 and 3 during experiment.

For the assessment of voluntary exercise, rats were housed individually in a cage containing a wheel (1.00 m circumference, TajhizGostar). Each exercising rat had a separate running wheel in its cage that allowed it to run voluntarily during 8 weeks of the study. This stainless-steel running wheel was equipped with a digital magnetic counter that was activated by wheel rotation and wheel revolutions were recorded daily. Then the running distance per day was calculated as the number of wheel revolutions each day. Rats with running distance lower than ~2000 m per day were eliminated before statistical analysis.^23^ Considering the excluding criteria (exercise below standard protocols) statistical analysis was performed for 7 animals in each group.

### Quantification of Akt and ERK1/2 in heart by ELISA

On the final day of experiment, rats were sacrificed under deep anesthesia with ketamine/xylazine (88/10 mg/kg, i.p.). Heart tissue immediately removed and washed with saline 0.9%. Tissue samples were weighted, homogenized in PBS (pH 7.2-7.4) and centrifuged for 20 min at the speed of 3000 rpm and 4ºC. Then supernatants were collected in new tube and Akt and ERK1/2 levels were measured using sandwich rat ELISA kits. The ELISA assay was performed according to the manufacturer's instructions. Akt protein activation by phosphorylation at serine residue 473 (P-Akt) and ERK1/2 phosphorylation (PT202/Y204) was assayed with ELISA (Akt: Cat. No. CK-E91385; Hangzhou Eastbiopharm Co., Ltd., Hangzhou, China. ERK1/2:Abcam Cambridge, UK) and normalized to the total protein concentration for each sample as determined by the Bradford assay.^24^

### Total RNA extraction, cDNA synthesis and real time PCR

Expression of miR-126 and miR-210 was assessed by qRT-PCR. Triplicate assays were performed for each RNA sample. MicroRNA was extracted from the heart tissue using the miRCURYTM RNA Isolation Kit (Exiqon, Denmark) according to the manufacturer's protocol. The procedure was performed based on spin column using a proprietary resin as a separation matrix for RNA from other cell components. RNA content and purity were measured at a wavelength of 260-280 nm using Nanodrop 1000 spectrophotometer (Thermo scientific, Wilmington DE 19810 *U*SA).

cDNA synthesis was done according to LNA universal RT miRNA PCR kit (Exiqon, Denmark). Briefly, total RNA containing microRNA was polyadenylated and cDNA was synthesized using a poly (T) primer with a 3' degenerate anchor and a 5' universal tag.

Syber Green qPCR Mix purchased from Exiqon (denmark) and used for real time PCR. Real time PCR was done using Rotor-Gene 6000 Corbett. The 2^-(ΔΔCt)^ method was used to determine relative quantitative levels of miR-126 and miR-210. The results were expressed as the fold-difference to the control group. Mir-1 was used as the endogenous control miRNA.

### Immunohistochemical assessments

For the investigation of angiogenesis in the heart tissue, samples from left ventricle were immersed into 10% formalin after excision, embedded in paraffin, and cut into 4 µm-thick slices. Sections were deparaffinized in xylene and dehydrated in a graded series of ethanol. Slides were incubated sequentially in proteinase K and treated by 0.3% hydrogen peroxide to block endogenous peroxidase activity. Sections were overlaid by primary antibody CD31 (Santa Cruz, USA) a marker of angiogenesis and incubated at +4ºC overnight. Sections were then washed and incubated with standard avidin-biotin complex (ABC; Santa Cruz) according to the manufacturer's instructions. Then, the slides were incubated in DAB (di-amino-benzidine, Santa Cruz), as the chromagen, and counterstained with Mayer's hematoxylin. Finally, sections were cleared in xylene, mounted with Entellan and assessed by light microscope (Olympus BX 40, Japan). Capillaries were visualized in the myocardium as a brown precipitate. Vascular structures positive for CD31 were counted for 5 to 6 slides per animal and 10 fields per slide.

### Statistical analysis

Results are presented as mean ± SD. Statistical analysis was performed using SPSS version 21.0 statistical software for Windows. All parameters were tested for normality using the one-sample Kolmogorov-Smirnov test. Average daily running distances for rats in each exercise group were averaged for each week and were compared using repeated measures ANOVA. Physical activity between Exe and Cro-Exe groups was analyzed using independent t-test. For Akt, ERK, CD31, miR-126, and miR-210 parameters, data were analyzed using two-way ANOVA followed by Tukey's post hoc test. A *p*-value less than 0.05 was considered statistically significant.

## Results

### Voluntary exercise

**Figure 1:**
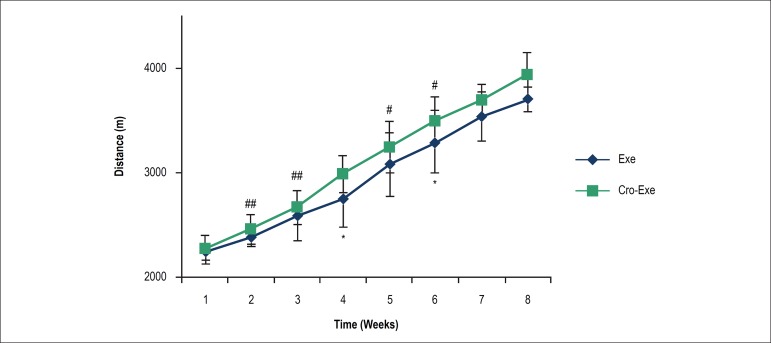
Running distance was averaged over each week of running wheel access (mean ± SD) for the 8-week duration of experiment, for both Exe and Cro-Exe groups (n = 7/group). There was no significant difference between groups. Animals in both Exe and Cro-Exe groups increased their average weekly running distance over the subsequent weeks. *p < 0.05 indicates a significant difference between consecutive weeks in Exe group. #p < 0.05 and ##p < 0.01 indicate significant differences between consecutive weeks in Cro-Exe group.

The Figure 1 illustrates the average running distance per week over the 8-week period of experiment. Animals ran voluntarily an average of 2.949 ± 178 m/week in the exercise group and an average of 3.090 ± 140 m/week in the crocin-exercise group. There was no significant difference in physical activity between Exe and Cro-Exe groups based on an independent t-test analysis.

Average running distance increased gradually in both Exe and Cro-Exe groups from first to eighth week. This increase in Exe group was significantly different from the prior week in fourth (p < 0.05) and sixth (p < 0.05) weeks. In Cro-Exe group, animals ran significantly more distance in second (p < 0.01), third (p < 0.01), fifth (p < 0.05), and sixth (p < 0.05) weeks than the prior week.

### Effects of crocin combined with voluntary exercise on Akt levels in the heart tissue

**Figure 2:**
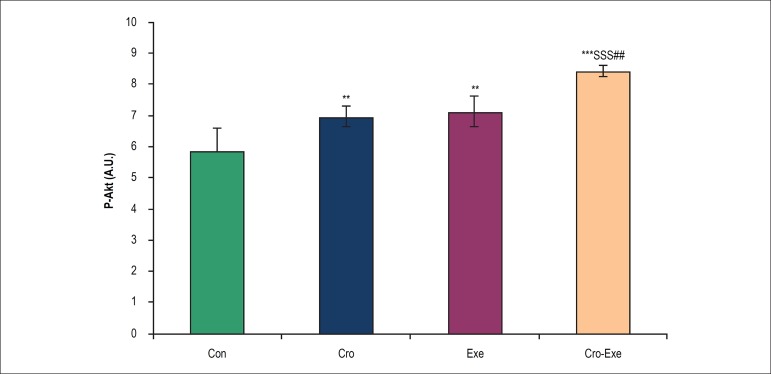
Effect of crocin and voluntary exercise on p-Akt levels. Data are shown as mean ± SD for n = 7 animals. ^***^p < 0.001 and ^**^p < 0.01 indicate significant differences with control group,^$$$^p < 0.001 indicates a significant difference with Cro group, and ^##^p < 0.01 indicates a significant difference with Exe group.

After 8 weeks of administration of crocin or performing voluntary exercise, the level of p-Akt increased significantly in Exe (p < 0.01), Cro (p < 0.01) and Cro-Exe (p < 0.001) groups in comparison with Con group (Figure 2). A comparison between the Cro-Exe group with Exe and Cro groups exhibited significant difference among these groups (p < 0.01 and p < 0.001, respectively).

### Effects of crocin combined with voluntary exercise on ERK1/2 levels in the heart tissue

**Figure 3:**
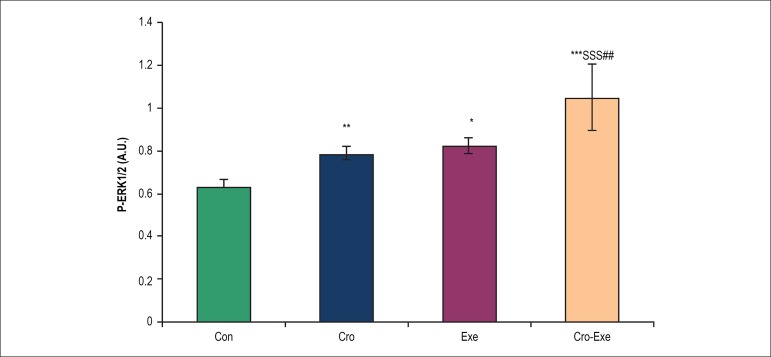
Effect of crocin and voluntary exercise on p-ERK1/2 levels. Data are shown as mean ± SD for n = 7 animals.^*^p < 0.05, ^**^p < 0.01, and ^***^p < 0.001 indicate significant differences with control group. ^$$$^p < 0.001 indicates a significant difference with Cro group and ^##^p < 0.01 indicates a significant difference with Exe group.

Two-way ANOVA showed that the p-ERK1/2 levels were significantly higher in rats treated with crocin or voluntary exercise than in control rats (Exe: p < 0.01, Cro: p < 0.05, and Cro-Exe: p < 0.001). Administration of crocin combined with exercise significantly increased p-ERK1/2 levels of the heart tissue compared to Exe (p < 0.01) and Cro (p < 0.001) groups (Figure 3). Figure 2 also indicates that crocin combined with voluntary exercise has a synergistic effect in p-ERK1/2 protein levels in heart tissue.

### Effects of crocin combined with voluntary exercise on miR-126 expression in the heart tissue

**Figure 4:**
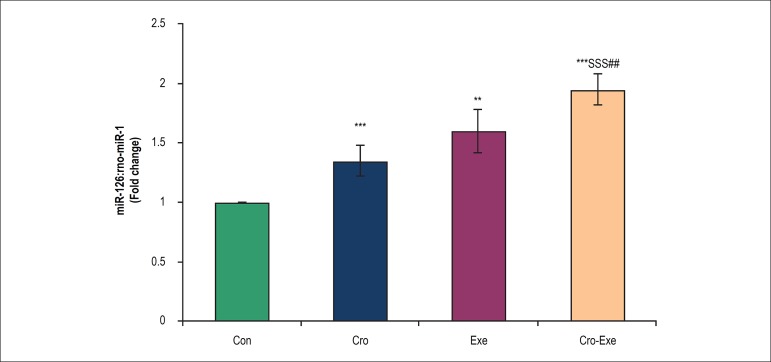
Effect of crocin and voluntary exercise on miR-126 expression levels. Data are shown as mean ± SD for n = 7 animals. ^**^p < 0.01 and ^***^p < 0.001 indicate significant differences with control group. ^$$$^p < 0.001 indicates a significant difference with Cro group and ^##^p < 0.01 indicates a significant difference with Exe group.

Two-way ANOVA showed that the miR-126 expression were significantly higher in rats treated with crocin (p < 0.001), voluntary exercise (p < 0.01) and crocin combination with exercise (p < 0.001) than in control rats. In the rats that underwent voluntary exercise and simultaneously received crocin for 8 weeks, expression of heart miR-126 significantly increased compared with Exe (p < 0.01), and Cro (p < 0.001) groups (Figure 4).

### Effects of crocin combined with voluntary exercise on miR-210 expression in the heart tissue

**Figure 5:**
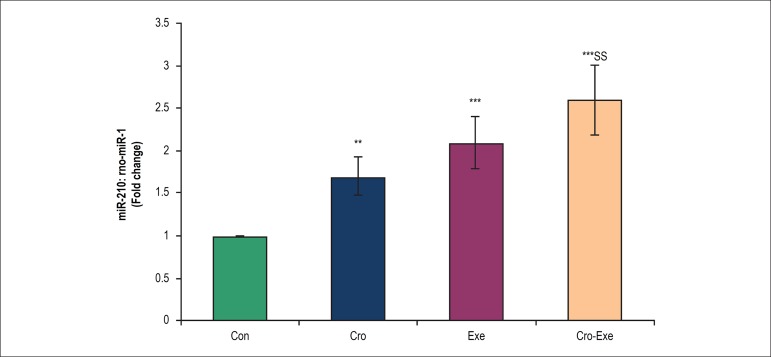
Effect of crocin and voluntary exercise on miR-210 expression levels. Data are shown as mean ± SD for n = 7 animals. ^**^p < 0.01 and ^***^p < 0.001 indicate significant differences with control group and ^$$$^p < 0.001 indicates a significant difference with Cro group.

As shown in Figure 5, following crocin administration and exercise performing, the heart expression level of miR-210 was significantly upregulated in Cro and Exe groups when compared to control group (p < 0.01 and p < 0.001, respectively). On the other hand, the expression of miR-210 increased significantly in Cro-Exe group compared with control group (p < 0.001). In addition, there is a significant difference between Cro-Exe and Cro groups (p < 0.01).

### Effects of crocin combined with voluntary exercise on CD31^+^ cells in myocardial capillary network

**Figure 6:**
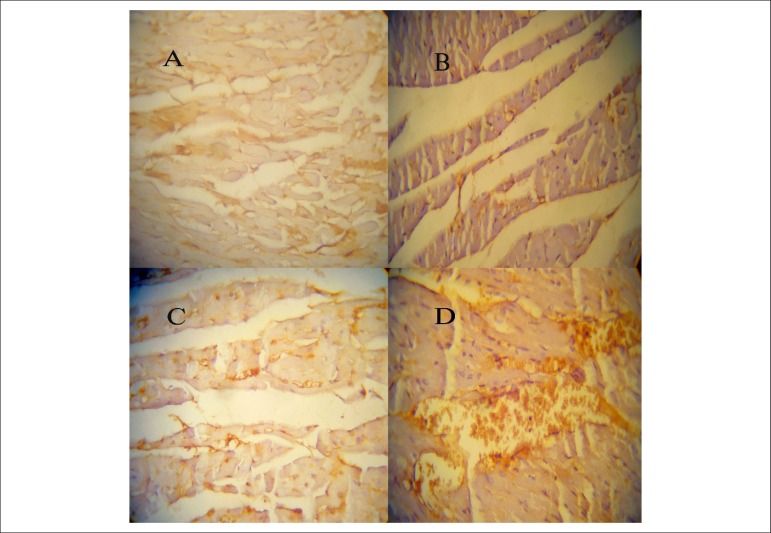
A) Representative images of CD31 staining (brown) in cardiac vessels of control, exercise, crocin and exercise-crocin groups (Magnification ×400). A: Con, B: Exe, C: Cro, D: Cro-Exe. B) Microvessel density was analyzed by immunohistochemistry for CD31. Microvessel density was quantified using 6 slides per animal and 10 fields per slide. Data are shown as mean ± SD for n = 7 animals. ***p < 0.001 indicates a significant difference with control group. ###p < 0.001 and $$$p < 0.001 indicate significant differences with exercise group and crocin group, respectively.

As demonstrated in figure 6, number of CD31^+^ cells were higher in animals that received crocin (p < 0.05) or performed exercise (p < 0.05) compared with control group (Figures 6A, B, C). In addition, CD31^+^ cells were significantly higher in sections from the heart of Cro-Exe group than Exe (p < 0.01) and Cro (p < 0.01) groups (Figure 6D). Thus, crocin combination with voluntary exercise appears to enhance vasculogenic response.

## Discussion

In the present study, we demonstrated that miR-126 and miR-210 expression of rat cardiac tissue increased in crocin, voluntary exercise, and exercise-crocin groups. In addition, crocin and voluntary exercise stimulated Akt and ERK1/2 proteins and angiogenesis in the heart tissue. For the first time, our study demonstrated that heart miR-126 and its related pathways including Akt and ERK1/2 upregulated in response to crocin combined with voluntary exercise in rats. Furthermore, our findings showed that crocin administration and voluntary exercise performing increased the expression of heart miR-210.

MiR-210, a hypoxia-specific miRNA, depends on HIF activation and upregulated after hypoxia.^25^ When miR-210 is overexpressed in endothelial cells, the ability of these cells to form blood vessels becomes pronounced, more than that of cells with normal levels of expression. Confirming miR-210 proangiogenic role, up-regulation of miR-210 in CD34+ cells increased tissue perfusion and capillary density in a mouse model of hind limb ischemia.^26^ Previous research has indicated that miR-210 may improve angiogenesis through the negative regulation of its target gene, ephrin A3, which is an important member of the ephrin angiogenesis regulatory gene family.^27^ Heart tissue expresses a variety of miRNAs, but little is known about the cardiac angiogenic response to crocin and exercise.^28^ Preclinical work demonstrated that intracardiac injections with a minicircle vector carrying miR-210 in a mouse model of myocardial infarction promoted significant improvement of left ventricular fractional shortening, decreased cellular apoptosis, and increased neovascularization.^9^ In the current study, we observed that crocin and voluntary exercise increased miR-210 expression levels in heart tissue using quantitative real-time PCR analysis. The miR-210 upregulation seems to be dependent on the exercise performing because the group that performed voluntary exercise showed a stronger miR-210 expression than crocin group. A probable mechanism is that during exercise, local hypoxic conditions in the cardiac muscle can occur and hypoxic situation trigger a number of physiological responses such as angiogenesis through HIF-1α-induced miR-210 expression.^30,31^ These data are in line with the observations made by Anja Bye et al.^32^ regarding significant increase in miR-210 expression in subjects with low Vo2max following the exercise activity.

MiR-126 is a pro-angiogenic miR, which is strongly expressed in the heart endothelium and directly targets SPRED1 and PIK3R2 for repression and functions to promote VEGF signaling.^14,15^ In fact, miR-126 activates survival kinases including ERK and Akt by downregulation of its targets and enhances the actions of VEGF.^16,17^ In endothelial cells, VEGF promotes angiogenesis through the phosphorylation of ERK1 and Akt. ERK and Akt are well known kinases that activate and promote cell proliferation by stimulating growth factors.^18^

In the present study, we showed that miR-126 regulates heart angiogenesis via Akt and ERK1/2 pathways in response to crocin and voluntary exercise. However, a few studies available say that exercise can increase miR expression in cardiac tissue. In line with our results, Uhlemann et al.^33^ reported that miR-126 expression increased after acute endurance exercise. Major findings also emerge from Fernandes et al.^34^ study indicating that exercise training restored the levels of peripheral miR-126 associated with revascularization in hypertension. Despite the observation that exercise affects endothelial function, the exact mechanism remains speculative. It is well established that exercise can increase VEGF levels, which is one of the major regulators of angiogenesis and cell survival.^35^ VEGF binds to VEGFR2 and promotes endothelial survival and angiogenesis signals which are intermediated by PI3K and its downstream target of the Akt and ERK1/2. In addition, we showed that ERK1/2 and Akt levels increased under high expression of miR-126. Therefore, it seems that voluntary exercise relieves the repressive influence of Spred-1/PI3K on the Akt and ERK1/2 by miR-126 overexpression, which finally improves cardiac angiogenesis. This finding is in agreement with a previous study that showed that PI3KR2 mRNA expression in the heart decreased in the exercise groups and it was associated with increase in protein expression of PI3K and phosphorylated Akt.^36^

In this study, we also showed that crocin regulates heart angiogenesis through miR-126 and its related Akt and ERK1/2 pathways. Crocin, a carotenoid pigment of saffron, has different pharmacological functions on the nervous,^37^ cardiac,^38^ and renal^39^ systems. Cardioprotective effects of crocin have been reported in some studies that are related to improvement of antioxidant activities and cardiac biomarkers.^3,40^ Although many researchers have explored the roles of crocin on different tissues, only a few studies have investigated the effects of crocin on angiogenesis in the heart tissue. Bie et al^41^ demonstrated that saffron increased expression of VEGF-R2 and promoted angiogenesis following brain injury in rats. Furthermore, it has been reported that the PI3K/Akt pathways are activated by crocin in the ganglion cell layer after retinal IR injury.^42^ Kang et al^43^ also showed that saffron increased the phosphorylation of mitogen-activated protein kinases (MAPKs), as one member of ERK family, in the muscle cells. Also our previous study confirms that crocin increases VEGF-A levels in the heart tissue of diabetic and non-diabetic rats.^40^

Based on the present results it could be concluded that crocin pretreatment improved cardiac angiogenesis, the effect which can be attributed to its ability of increasing Akt and ERK1/2 levels via enhancement of miR-126. Preservation of histoarchitecture of heart tissue by crocin pretreatment confirms these effects. Regarding the limitations of this study, we did not measure other factors involved in angiogenesis and we referred to previous studies. Further studies are needed to explore other possible mechanisms and pathways that might be directly or indirectly involved in its cardioprotective effects. Therefore, we suggest that crocin by increasing of miR-126 and enhancement of VEGF signaling pathways through Akt and ERK1/2 can induce cardiac capillary formation. In addition, we showed that crocin combination with voluntary exercise has synergistic effects on miR-126 expression and Akt, ERK1/2 levels in heart tissue, which was the first study in rat cardiac angiogenesis.

## Conclusion

This study shows that crocin in combination with voluntary exercise promotes cardiac angiogenesis and this may be related to expression of miRNA-126 and miR-210. Further studies about the mechanism of crocin and voluntary exercise on cardiac angiogenesis may provide a basis for the development of new therapeutic or preventive approaches to some overcome cardiovascular diseases.
